# Effects of Carpal Tunnel Syndrome on Dexterous Manipulation Are Grip Type-Dependent

**DOI:** 10.1371/journal.pone.0053751

**Published:** 2013-01-10

**Authors:** Wei Zhang, Jamie A. Johnston, Mark A. Ross, Kyle Sanniec, Elizabeth A. Gleason, Amylou C. Dueck, Marco Santello

**Affiliations:** 1 School of Biological and Health Systems Engineering, Arizona State University, Tempe, Arizona, United States of America; 2 Department of Physical Therapy, College of Staten Island, City University of New York, Staten Island, New York, United States of America; 3 Faculty of Kinesiology and Hotchkiss Brain Institute, University of Calgary, Calgary, Canada; 4 Mayo Clinic Hospital, Phoenix, Arizona, United States of America; The University of Western Ontario, Canada

## Abstract

Carpal tunnel syndrome (CTS) impairs sensation of a subset of digits. Although the effects of CTS on manipulation performed with CTS-affected digits have been studied using precision grip tasks, the extent to which CTS affects multi-digit force coordination has only recently been studied. Whole-hand manipulation studies have shown that CTS patients retain the ability to modulate multi-digit forces to object mass, mass distribution, and texture. However, CTS results in sensorimotor deficits relative to healthy controls, including significantly larger grip force and lower ability to balance the torques generated by the digits. Here we investigated the effects of CTS on multi-digit force modulation to object weight when manipulating an object with a variable number of fingers. We hypothesized that CTS patients would be able to modulate digit forces to object weight. However, as different grip types involve the exclusive use of CTS-affected digits (‘uniform’ grips) or a combination of CTS-affected and non-affected digits (‘mixed’ grips), we addressed the question of whether ‘mixed’ grips would reduce or worsen CTS-induced force coordination deficits. The former scenario would be due to adding digits with intact tactile feedback, whereas the latter scenario might occur due to a potentially greater challenge for the central nervous system of integrating ‘noisy’ and intact tactile feedback. CTS patients learned multi-digit force modulation to object weight regardless of grip type. Although controls exerted the same total grip force across all grip types, patients exerted significantly larger grip force than controls but only for manipulations with four and five digits. Importantly, this effect was due to CTS patients’ inability to change the finger force distribution when adding the ring and little fingers. These findings suggest that CTS primarily challenges sensorimotor integration processes for dexterous manipulation underlying the coordination of CTS-affected and non-affected digits.

## Introduction

Carpal Tunnel Syndrome (CTS), a compression neuropathy of the median nerve, is one of the most common diseases affecting hand function. The median nerve is a mixed nerve comprised of sensory and motor axons innervating most extrinsic hand flexor muscles and some intrinsic muscles, and relays sensory information from the palmar aspect of the thumb, index, middle and the lateral half of the ring finger. Carpal Tunnel Syndrome leads to impairment in hand sensorimotor function that starts with loss of sensation in the palmar and the most distal dorsal aspect of thumb, index, middle, and lateral half of the ring finger. Note that CTS patients have intact sensation on the medial half of the ring finger and the little finger as these are innervated by ulnar nerve. In severe cases, CTS can also affect motor fibers thus leading to force deficits mostly in the thumb. From a behavioral standpoint, CTS patients often report loss of manual dexterity, such as difficulties with fine manipulation or dropping objects. Despite the widespread incidence of CTS (3.7% in the general population of the U.S. [1]) and its impact on activities of daily living, relatively little research has been performed on the effects of median nerve compression on the control of dexterous manipulation.

Early work on the effects of CTS on two-digit manipulation (i.e., precision grip performed with thumb and index finger) has reported conflicting evidence. For example, it has been reported that CTS patients exert larger grip force than healthy controls when applying dynamic forces with a tool [2]. Studies of experimental models of CTS in healthy subjects using mechanical compression of the median nerve [3], injection of anesthesia into the carpal tunnel [4], and digit local anesthesia [5], [6], have also reported increased normal force amplitude relative to control conditions. In contrast, other studies have reported that grip force in CTS was similar to controls during point-to-point arm movements of an object held with a whole-hand grip [7] or no effects of CTS on patients’ ability to modulate grip force to texture using a two-digit grip [8]. However, several factors prevent a direct comparison among these studies, including differences in the tasks used (e.g., grasp to lift vs. point-to-point movements) and the number of digits studied, i.e., CTS-affected digits only (two-digit grip) or CTS-affected and non-affected digits (whole-hand grip).

The extent to which CTS affects the modulation of multi-digit forces (whole-hand grasp) to object properties has only recently been investigated. These studies revealed that CTS patients are able to manipulate objects by scaling multi-digit forces to object weight [9], mass distribution [10], and frictional properties [11], thus effectively preventing object slip or tilt. This ability could be attributed to residual tactile sensation in the CTS-affected digits and/or proprioceptive inputs from forearm and arm muscles signaling object weight. However, CTS patients also exhibited sensorimotor deficits relative to healthy controls, including significantly larger grip force and lower ability to balance the torques generated by the digits. As the median nerve compression spares sensation of the little finger and the medial half of the ring finger, these deficits could be due to sub-optimal integration of intact sensation from these digits with impaired sensation from CTS-affected digits. This interpretation is consistent with the observation that when only CTS-affected digits (thumb and index finger) are used to grasp and lift an object, CTS patients exert similar grip force as controls [8]. However, conflicting evidence of larger grip force in two-digit manipulation in CTS [2], compounded by the fact that different studies used patients with heterogeneous levels of CTS severities, warranted a systematic investigation of the interaction between grip type and CTS on the control of dexterous manipulation.

The purpose of the present study was to quantify the effects of CTS on sensorimotor integration underlying the coordination of digit forces for manipulating objects with different weights when using a variable number of digits. This work extends our previous work on CTS that focused on the control of manipulation using only a five-digit grip configuration [12]. Based on this previous study, we hypothesized that CTS patients would maintain the ability to modulate digit forces to object weight regardless of grip-type. As different grip types involve the exclusive use of CTS-affected digits (‘uniform grip’: two- and three-digit grips) or a combination of CTS-affected and non-affected digits (‘mixed grip’: four- and five-digit grips), we envisioned two alternative outcomes: (1) the above CTS-induced coordination deficits will be greater for ‘mixed’ than ‘uniform’ grips due to the potentially more challenging task of coordinating multi-digit forces based on the integration of feedback from CTS-affected and non-affected digits; or CTS-induced coordination deficits described for five-digit grasping [9]–[11] will be the same for ‘mixed’ and ‘uniform’ grips, indicating no effect of using exclusively CTS-affected digits or combining them with CTS-non affected digits.

## Methods

### Ethics Statement

All participants were naïve to the purpose of the study and gave their written informed consent according to the declaration of Helsinki. The experimental protocols were approved by the Institutional Review Boards at Arizona State University and Mayo Clinic Hospital.

### Subjects

Sixteen Carpal Tunnel Syndrome (CTS) patients (3 males and 13 females; mean ± standard deviation: 45±3 years old; average weight and height: 83.7±8.8 kg and 166.5±3 cm respectively) and sixteen age-, gender- and handedness-matched healthy controls (average weight and height: 77.7±5.2 kg and 167.2±2.5 cm, respectively) participated as subjects in the study. CTS diagnosis was performed by the same neurologist (Mayo Clinic Hospital, Phoenix, AZ) based on clinical symptoms and results of electrodiagnostic tests (Table 1; normative values are shown in Table 2). Inclusion and exclusion criteria for CTS patients and controls were the same as reported in our previous study [9]–[10]. Specifically, for inclusion in our study, CTS patients had to exhibit at minimum a prolonged median nerve distal sensory latency (antidromic or orthodromic, relative or absolute). For controls, eligibility for participation to our study included absence of CTS-like symptoms as verified by testing using Semmes-Weinstein monofilaments and provocative tests (Durkan’s nerve compression and Phalen’s and Tinel’s tests). The neurologist carefully reviewed the detailed clinical history of CTS patients and controls. We included only patients with idiopathic mild and moderate CTS in the study and excluded patients with categories of severe or markedly severe CTS who are most likely to have impaired motor function of the hand. Specifically, all of the patients had predominantly sensory symptoms (e.g., pain, tingling, and numbness) leading to their evaluation. Note that the patients’ touch sense on the median nerve innervated digits was affected by CTS (e.g., parasthesia and a slightly dulled sense of touch), but the digits were not totally numb. No patient had motor deficits identifiable as weakness or muscle atrophy on physical examination, despite some of them having a prolonged motor wrist latency suggesting the motor nerve fibers are affected by the disease process. In addition, all patients had normal values for motor amplitude (see Table 1).

**Table 1:** Subjects’ Basic Information and CTS Patient’s Results of Electrodiagnostic Tests.

No.	CTS Patients									Control
	Gender	Age	Handedness	Testedhand	Electrodiagnostic test results (abnormal values in bold)^1^					Age
					Median NerveStudy	Amplitude^2^	Velocity^3^ (m/s)	Distallatency (ms)	F-waveLatency^4^ (ms)	
1	F	36	R	R	Sensory	**7.3**		**3.7**		36
					Motor	7.3		**5.4**	28.0	
2	F	57	R	R	Sensory	**16.9**	57	**3.1**		57
					Motor	8.7		**4.7**	28.5	
3	F	51	R	R	Sensory	**15.8**		**3.5**		53
					Motor	10.9	46	**4.8**		
4	F	28	R	R	Sensory	**12.7**	65	**2.7**		29
					Motor	4.1	57	4.1	25.0	
5	F	52	R	L	Sensory	74.0	67	**2.5**		50
					Motor	4.7	55	**4.8**	27.8	
6	M	51	R	L	Sensory	**46.7**		**2.6**		50
					Motor	9.8	51	4.2		
7	F	59	R	R	Sensory	65.0	64	**2.4**		59
					Motor	9.4	56	4.2	24.2	
8	F	31	R	R	Sensory	**22.2**		**3.2**		31
					Motor	9.0	55	**4.7**		
9	F	54	R	R	Sensory	90.0	65	**2.4**		54
					Motor	7.9	53	4.1	26.5	
10	F	33	R	R	Sensory	50.4	63	**3.3**		32
					Motor	6.6	54	**4.9**	29.6	
11	M	32	R	L	Sensory	**8.1**	56	**4.0**		33
					Motor	7.7	55	**5.5**	33.0	
12	F	59	R	R	Sensory	77.3	60	**2.5**		60
					Motor	7.4	56	**4.6**	27.8	
13	F	56	R	R	Sensory	50.7		**2.9**		56
					Motor	6.3	58	4.7		
14	M	22	L	R	Sensory	87.9		**2.5**		23
					Motor	16.6	59	4.3		
15	F	46	R	R	Sensory	**30.2**	63	**2.8**		47
					Motor	10.5	52	**4.9**	27.8	
16	F	54	R	R	Sensory	6.5		**3.6**		56
					Motor	6.2	50	**5.4**		

1 Normative values are listed in Table 2. Sensory studies are orthodromic.

2 Amplitude values for sensory studies are microvolts and motor studies are millivolts.

3,4 Conduction velocities and F-wave latencies were normal for all nerve studies.

**Table 2:** Normative Median and Ulnar Nerve Conduction Values, Mayo Clinic Arizona EMG laboratory.

Nerve	Age <60		Age ≥60^2^	
Median	Amplitude^1^	Wrist latency (ms)	Amplitude^1^	Wrist latency (ms)
Orthodromic sensory	≥50	<2.3	M ≥17.4; F ≥40.1	<2.5
Antidromic sensory	≥15	<3.5	M ≥12.2; F ≥15.9	<3.7
Motor	≥4	<4.5	≥4.5	M: <4.4; F <3.8
**Ulnar**				
Orthodromic sensory	≥15	≤2.3	M ≥3.4; F ≥14.4	<2.3
Antidromic sensory	≥10	<3.1	M ≥3.9; F ≥15.9	M <3.5; F <3.1
Motor	≥6	<3.6	≥4.8	M: <3.2; F <2.9

1 Amplitude values for sensory studies are microvolts and motor studies are millivolts.

2 Note that some normal values for subjects 60 years old and older are gender specific. M = male; F = female.

All but one CTS patient and control were right-handed (self-reported). Three CTS patients were tested on their left hand and thirteen patients were tested on their right hand. The tested hand of control subjects was matched to the hand tested in CTS patients.

### Apparatus

**Figure 1:**
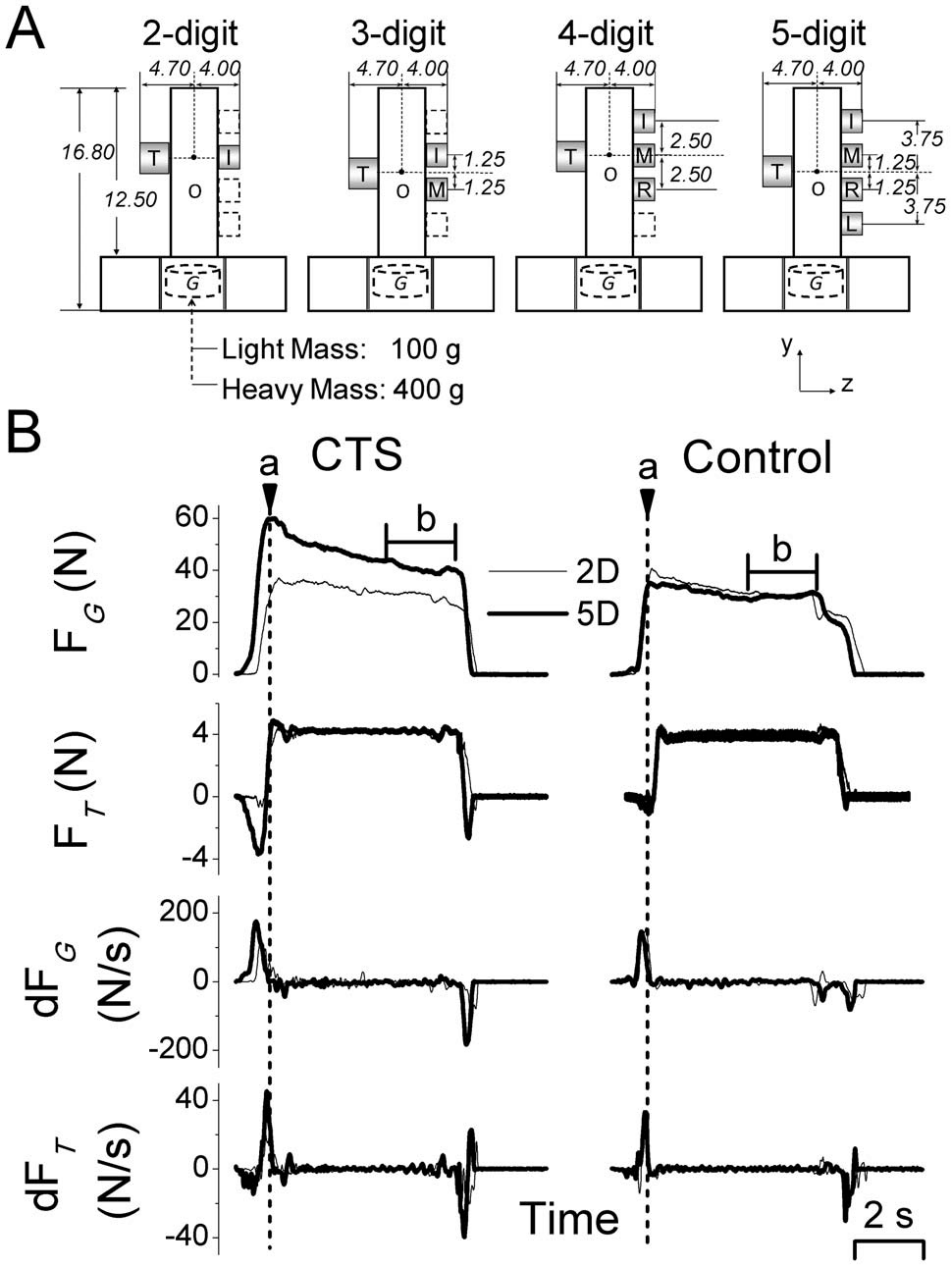
Grip device and experimental variables. Panel A shows the front view of the grip device used for each grip type condition. All dimensions shown are in cm. Force/torque (F/T) sensors are mounted on both sides of the device to measure forces and moment of forces exerted by each digit involved in a given grip type condition (thumb, index, middle, ring, and little fingers: T, I, M, R, and L, respectively). A mass (*“G”*; 100 g or 400 g) was inserted in the midpoint at the bottom of the grip device (“Light mass” and “Heavy mass”, respectively). For the 2- and 4-digit grip types, the center of the thumb sensor was collinear with the center of the index or middle finger sensor, respectively. For the 3- and 5-digit grip types, the center of the thumb sensor was collinear with the mid-point between the centers of the index and middle finger or middle and ring finger sensors, respectively. Note that for each grip type, all five sensors were mounted on the grip device to maintain a constant mass for a given mass condition, thus changes in grip types were implemented by changing the relative position of F/T sensors. A magnetic tracker (not shown) was used to track the object position and orientation of the object during the manipulation. ‘O’ denotes the point about which moments were computed (see Methods for more details). Panel B shows, from top to bottom, the time course of the sum of all digit grip forces (F*_G_*), the sum of all digit tangential forces (F*_T_*), and the derivatives of F*_G_* and F*_T_*. Data are aligned with object lift onset (vertical dashed line, a). Note that analysis of digit forces during object hold was performed on data averaged over the last 2 seconds of the hold (horizontal bar, b). Data are from one representative CTS patient (S3) and her matched control (left and right column, respectively) performing the task on the seventh trial for the “light mass” condition and two grip types (two- and five-digit, 2D and 5D, respectively).

The grip device used in our study is shown in Figure 1A. Five force/torque (F/T) transducers (oneNano-25 for thumb and four Nano-17 for other digits, ATI Industrial Automation, Apex, NC) mounted on the vertical bar of the device were used to measure the forces and torques produced by individual digits (T, thumb; I, M, R, and L denote index, middle, ring, and little fingers, respectively). The surface of each sensor was covered with insulating circular plates. An electromagnetic position/orientation tracking sensor (Polhemus Fastrak, Colchester, VT; 0.075 mm and 0.05° resolution) was mounted on the top of the grip device to measure the object position and angle about the vertical axis in the frontal plane, i.e., object roll.

The vertical location of the F/T sensor for the thumb was adjusted such that the center of the thumb sensor was always aligned with the center of one sensor or the midpoint between the centers of two or more sensors depending on the number of digits involved in the grasp. Specifically, when subjects were instructed to use two digits (2D) with T and I, three digits (3D) with T, I, and M, four digits (4D) with T, I, M, and R, or five digits (5D) with T, I, M, R, and L, the center of the T sensor was aligned with the center of the I sensor, the midpoint between the I and M sensors, the center of the M sensor, or the midpoint between the M and R sensors, respectively (Fig. 1A). We studied two mass conditions: ‘light mass’ (total mass: 445 g) and ‘heavy mass’ (total mass: 745 g), by adding a mass of 100 g or 400 g, respectively, in the midpoint at the bottom of the grip device (Fig. 1A). Note the added mass was not visible to the subjects during the experiment. Force and torque data from each sensor were acquired by five 12-bit A/D converter boards (National Instruments, Austin, TX) at a sampling frequency of 1 kHz. Collection of position data was triggered by the onset of force data acquisition and collected on a separate computer at a sampling frequency of 80 Hz. Force and position data were synchronized offline for analyses. Custom software (LabVIEW 6.1, National Instruments) was used to acquire, display and store force data.

### Experimental Procedures

During the experiment, subjects sat facing the grip device with the shoulder of the tested hand aligned with the grip device to ensure that the object could be comfortably grasped. Subjects were instructed to reach, grasp, lift ∼10 cm from the table, hold for 4 s, and replace the grip device on the table at a comfortable, self-selected pace. One of the experimenters visually verified that the subject contacted each sensor with the tip of a single digit. The only task requirement was to lift and hold the grip device vertically.

Subjects were asked to use one of four grip types to complete the above-described task: two digits (2D: thumb and index finger), three digits (3D: thumb, index, and middle fingers), four digits (4D: thumb, index, middle, and ring fingers), and five digits (5D). Note that for each grip type, all five sensors were mounted on the grip device to maintain a constant mass and mass distribution for each “mass” condition. Therefore, changes in grip type were implemented by changing the relative position of the thumb F/T sensor. For each grip type and “mass” condition, subjects were instructed to grasp and lift the device for seven consecutive trials. Thus, each subject performed a total of 56 trials (7 trials × 2 conditions × 4 grip types). Note that subjects were unaware of object mass on the first trial of each “mass” condition, but were aware that the load would remain the same within each block of seven trials. Grip types were presented in a counterbalanced order across CTS patients, and two “mass” conditions were presented in a pseudo-randomized order within each grip type block. The order of grip type and object mass presentation was matched between each CTS patient and his/her control. Subjects were given a minimum of 10-s rest period between trials and experimental conditions to prevent pain, fatigue, or worsening of the CTS symptoms. None of our subjects reported any of these adverse reactions.

### Data Processing

Force and position data were temporally aligned offline and analyses were performed using MATLAB 7.12 (MathWorks), Excel 2007 (Microsoft), Minitab 15 (Minitab), and SPSS version 19 (SPSS). Experiment variables were analyzed at two time phases of the grasp (Fig. 1B): (1) object *lift onset* and (2) object *hold*. As we described in our previous work [9]–[10], object lift onset was used to examine anticipatory scaling of digit forces and total moment to object mass based on previous manipulations, whereas object hold was used to evaluate subjects’ ability to adapt digit forces as a result of sensory feedback acquired following object lift onset. Briefly, object lift onset was defined as the time at which the grip device vertical position crossed and remained above a threshold (mean +2 SD of the signal baseline) for 200 ms, and object hold was defined as the time period between the end of object lift and the onset of object replacement on the table. These two events were defined as the time at which the derivative of object vertical position dropped less than 3% of its maximal value during object lifting and decreased lower than 3% of its minimum value during object release, respectively. As object hold duration varies across lifts and subjects, and because force transients occur at the onset of object hold, digit forces were averaged over the last 2 s of the object hold phase. We analyzed the following experimental variables:

1. *Digit forces.* Digit *normal force* (F*n*) is the force component perpendicular to the grip surface (Fig. 1B). Grip force (F*_G_*) was defined as the sum of F*n* produced by all digits. Digit *tangential force* (F*tan*) is the vertical force component parallel to the grip surface produced by each digit to lift and hold the object against gravity. Total tangential force (F*_T_*) was defined as the sum of F*tan* produced by all digits (Fig. 1B).
2. *Coefficient of variation of digit force.* We quantified across-trial variability in force control as the coefficient of variation of F*n* exerted during object hold (CV_F*n*) for each finger (I, M, R, L) across the last five trials (Trial 3–7).
3. *Derivative of digit forces.* We computed the derivative of F*_G_* (dF*_G_*) and analyzed *peak rate of F_G_* occurring between digit contact and object lift onset. This variable was used to assess anticipatory control of digit force to object mass [13].
4. *Digit moment of forces*. Digit moment of force (referred as ‘moment’ hereafter) was defined as the sum of the moments exerted by the digit(s) in the frontal plane (*yz* plane) about the origin ‘O’ (Fig. 1A). Moment analysis was used to quantify the extent to which subjects could generate a moment at object lift onset in the direction opposite to the external moment caused by the load [12], [14]. Therefore, the current task requirement of lifting the object vertically while preventing it from rolling is fulfilled when the moment generated on the object is zero. The moment generated by the subjects at object lift onset was defined as *compensatory moment* (M*com*) and was computed as the resultant moment produced by all the digits’ normal forces (normal moment; M*n*) and digits tangential forces (tangential moment; M*tan*) [9], [12]. For M*com* to be zero as required by our task, M*n* should cancel M*tan* at object lift onset, i.e, they can either be both zero, or if one of them is nonzero, these two components should covary negatively.
5. *Object roll.* The current task required subjects to minimize object roll during object lift and object hold. Thus, *peak object roll* [12], [15]–[16] was used as a performance measure to further quantify the extent to which both subject groups could implement anticipatory grasp control.
6. *Time intervals*. To quantify the temporal aspects of multi-digit forces force development, we analyzed the following three time intervals: *(a)* from first to last digit contact on their respective sensors (*t_CF_CL_*), *(b)* from last digit contact to peak rate of F*_G_* (*t_CL_PRF_*), and *(c)* from last digit contact to object lift onset (*t_CL_LON_*).

### Statistical Analysis

To determine the extent to which the effects of CTS, grip type, and mass on multi-digit force coordination changed as a function of consecutive lifts, we first performed 4-way analysis of variance (ANOVA) with repeated measures on all of the above described experiment variables with *Group* (two levels: CTS and controls) as between-subject factor, and *Grip type* (four levels: 2D, 3D, 4D and 5D), *Mass* (two levels: light and heavy), and *Trial* (seven levels: 1^st^ through 7^th^ trials) as within-subject factors. Consistent with our previous CTS work [9], multi-digit forces and peak object roll changed as a function of trial, however, the only significant changes occurred during the first couple of trials. Specifically, after the third trial subjects fully adapted multi-digit forces to object mass (post-hoc tests: no significant differences across trials in digit forces or peak object roll from trial 3 through 7; *P*>0.05). Furthermore, this analysis revealed no significant interaction between *Trial* and either *Group* or *Grip type* for any experimental variable. As the focus of the present paper was on whether CTS affects multi-digit force coordination as a function of grip type, for all subsequent analyses we omitted the factor *Trial* and averaged data across trials 3 to 7.

We performed 3-way analysis of variance (ANOVA) with repeated measures with *Grip type* and *Mass* as within-subject factors and *Group* as the between-subject factor on five primary variables: *(a)* object peak roll during lift, *(b)* grip force (F*_G_*) at object lift onset and hold (separate ANOVA’s), and *(c)* absolute values of M*com*, M*n*, and M*tan* at object lift onset. The same statistical design was also used on *(d)* peak rate of F*_G_* to assess anticipatory force control and *(e)* the above-described time intervals.

To evaluate the effects of grip type and CTS, additional analysis was performed on *individual* finger forces and their across-trial variability (note that the above analysis was performed on the net forces or moments exerted by all digits combined). This analysis consisted of 3-way ANOVAs with repeated measures with *Grip type* and *Mass* as within-subject factors, and *Group* as the between-subject factor on *(a)* F*n*, *(b)* F*tan* and *(c)* CV_F*n* at object lift onset as well as at object hold separately for each finger that was involved in at least two different grip types (i.e., I, M, R). Note that the number of levels for the factor *Grip type* differed depending on the finger examined as the number of instances a given finger was involved in each grip type changed, i.e., four levels for the index finger, three levels for the middle finger, and two levels for the ring finger. Lastly, we also performed 2-way ANOVAs with repeated measures on the above variables for the little finger at object lift onset and hold separately, with *Mass* as within-subject factors and *Group* as the between-subject factor. This was motivated by the fact that the little finger was only employed in the 5D grip, preventing the analysis of the factor *Grip type*.

Mauchly’s test was used to test for sphericity. When sphericity assumption was violated, Greenhouse-Geisser correction was used as an alternative method. Note that the reported *P*-value(s) associated with the F statistic(s) are adjusted via Greenhouse-Geisser. When appropriate, we performed post hoc pairwise comparisons with Bonferroni adjustments. A significance level of *P*<0.05 was used for all comparisons. All values reported in the text are averages of all subjects ± standard error of the mean.

## Results

All CTS patients and control subjects successfully completed our object grasp, lift, hold, and replace task without slipping or dropping the object while attempting to minimize object roll as instructed. As expected based upon our previous studies [9]–[10], both groups of subjects learned to perform our task after 1–2 trials after experiencing light or heavy object mass, i.e., no significant trial-to-trial difference from trial 3 to 7 (last five trials). Thus we omit results from trial-to-trial adaptation and focus on learned multi-digit force coordination over the last five trials.

### Manipulation Performance: Object Roll

The largest peak object roll (2.1±0.1°) occurred on the first object lift and slightly decreased during the last five trials in both groups (1.6±0.1°) regardless of object mass and grip type (no significant main effect of *Group* or significant interactions *Group* × *Mass* or *Grip* × *type*). Both groups exhibited the largest peak object roll when using a 2-digit grip type (main effect of *Grip type*: F_[3,90]_ = 6.41, *P*<0.001; peak object roll from 2D >4D and 5D).

### Time Intervals of Multi-digit Force Development

The temporal development of multi-digit forces was affected by grip type and object weight similarly across patients and controls for most time intervals. For both groups, time from last contact to *(a)* peak F*_G_* rate (*t_CL_PRF_*) and *(b)* object lift onset (*t_CL_LON_*) was longer when lifting a heavier object and when the grasp involved more digits. When lifting a heavier object, *t_CL_PRF_* and *t_CL_LON_* increased 28±10 ms (13%) (F_[1,30]_ = 6.48, *P*<0.05) and 82.9±17 ms (15%) (F_[1,30]_ = 22.48, *P*<0.001), respectively. For the effect of grip type, 2D and 5D were characterized by the shortest and longest intervals in both groups, respectively. Specifically, the average time between the first and last contact (*t_CF_CL_*) increased from 199±34 ms to 706±105 ms, *t_CL_PRF_* increased from 377±26 ms to 477±50.6 ms, and *t_CL_LON_* increased from 627±56.9 ms to 783±79.7 ms (main effect of *Grip type;* F_[3,90]_ = 24.42, F_[1.791,90]_ = 7.27, *and* F_[3,90]_ = 4.86, respectively; all *P*<0.001). However, after establishing contact with all digits, CTS patients developed grip force quicker than controls (main effect of *Group* on *t_CL_PRF_*: F_[1,30]_ = 4.39, *P*<0.05).

### Modulation of Grip Force as a Function of Object Weight and Grip Type

CTS patients exhibited anticipatory control of grip force similarly to controls, but exerted larger grip force than controls when using grips involving more digits. Both groups of subjects exhibited anticipatory control of grip force (F*_G_*) as revealed by larger peak F*_G_* rate when lifting the heavier object (114±8 N/s and 144±10 N/s for light and heavy mass, respectively) (main effect of *Mass*: F_[1,30]_ = 67.99, *P*<0.001). However, CTS patients produced larger peak F*_G_* rates (150±13 N/s) than controls (109±11 N/s) (main effect of *Group*: F_[1,30]_ = 5.96, *P*<0.05; Fig. 1B), especially for grip types with larger number of digits (*Group × Grip type* interaction: F_[3,90]_ = 2.78, *P*<0.05; posthocs showed larger peak F*_G_* rates in CTS patients for 4D and 5D but not in 2D and 3D). The larger F*_G_* rates in CTS patients were due to their larger F*_G_* at object lift onset (below).

**Figure 2:**
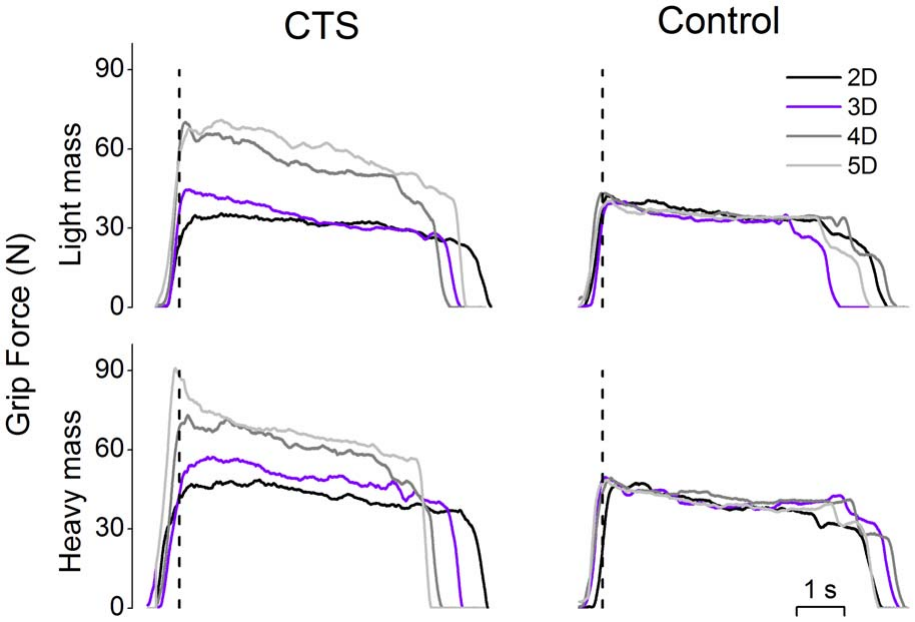
Time course of grip force as a function of grip type in CTS and controls. The time course of grip force (F*_G_*) from contact to object release is shown for a representative CTS patient (S3) and her control subject (left and right column, respectively) for the light and heavy mass conditions (top and bottom row, respectively). Data are from the last trial of each block performed with each grip type (two-, three-, four- and five-digit grasps are denoted by 2D, 3D, 4D, and 5D, respectively) and are aligned relative to object lift onset (vertical dashed line).

Figure 2 shows the time course of the total grip force (F*_G_*) exerted on the last trial of each trial sequence for each grip type for both object weights from a representative CTS patient and her matched control. Regardless of object weight, the CTS patient exerted larger F*_G_* before lifting the object throughout the lift and hold when using 4D and 5D grip types than 2D and 3D. In contrast, the control subject did not modulate F*_G_* when using different grip types to manipulate the object with a given weight.

**Figure 3:**
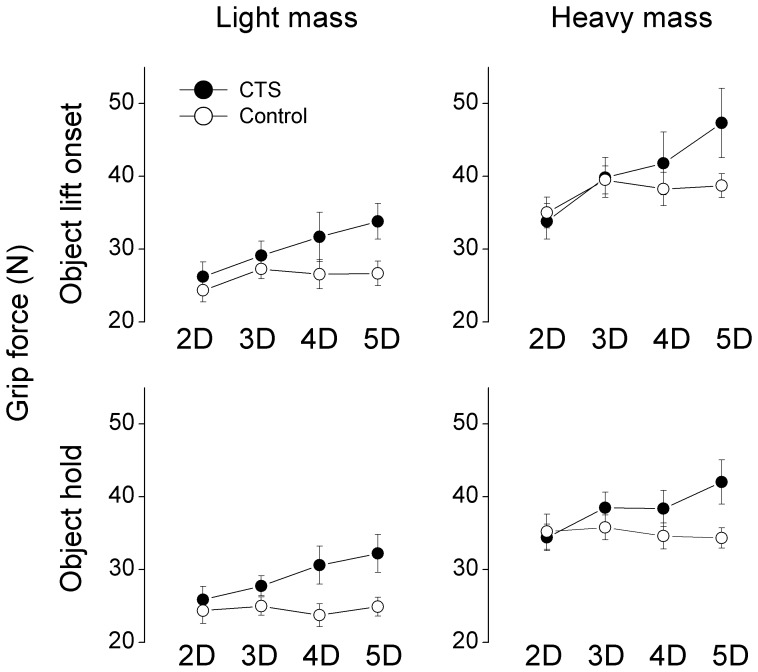
Grip force at object lift onset and during object hold. Grip force (F*_G_*) measured at object lift onset and during object hold (top and bottom row, respectively) is shown for each subject group and grip type. Data are mean values averaged across trials 3 through 7 for each subject group and mass condition (left and right column). Vertical bars denote standard errors.

The results shown in Figure 2 were common to all subjects (Fig. 3). ANOVA revealed that both subject groups exhibited F*_G_* modulation to object weight at object lift onset and during object hold (from 28.5±1.4 N for light mass to 39.2±1.8 N for heavy mass at object lift onset, and from 26.8±1.2 N for light mass to 36.7±1.4 N for heavy mass during object hold) (main effect of *Mass*: F_[1,30]_ = 357.54 and 197.04, respectively; both *P*<0.001). Grip types characterized by larger number of digits were associated with larger F*_G_* (main effect of *Grip type*: F_[3,90]_ = 8.82 for object lift onset; F_[3,90]_ = 4.03 for object hold, both *P*<0.001). Most importantly, however, this tendency was stronger for CTS patients than controls (Fig. 3). Specifically, CTS patients exerted larger F*_G_* than controls for 4D (36.7±3.8 N and 32.4±2 N in CTS and controls at lift onset respectively; 34.5±2.4 N and 29.2±1.6 N in CTS and controls during object hold, respectively) and 5D grip types (41.7±4.1 N and 32.7±1.6 N in CTS and controls at object lift onset, respectively; 37.1±2.8 N and 29.6±1.3 N in CTS and controls during object hold, respectively), but not the 2D (30±2.2 N and 29.7±1.7 N in CTS and controls at object lift onset, respectively; 30.1±1.8 N and 29.8±2.1 N in CTS and controls during object hold, respectively) and 3D (34.5±2.3 N and 33.4±1.5 N in CTS and controls at object lift onset, respectively; 33.1±1.7 N and 30.4±1.4 N in CTS and controls during object hold, respectively) grip types regardless of object weight (*Group* × *Grip type* interaction; F_[3,90]_ = 3.74 for object lift onset, *P*<0.05; F_[3,90]_ = 4.97 for object hold, *P*<0.01).

### Finger Normal Force

There are two possible explanations for the above interaction between group and grip type that showed larger F*_G_* in patients than controls for 4D and 5D grip types: 1) the forces produced by the index and middle fingers remain similar between groups regardless of the grip type, but the patients produce larger forces than the controls in the ring and little fingers during the 4D and 5D tasks, or 2) the discrepancy in F*_G_* between the groups is due to not only the additional force produced by the ring and little fingers, but also to changes that occur in the forces exerted by the patient group in the thumb, index, and middle fingers. To distinguish between these two possibilities, we examined F_G_ distribution among the digits for each grip type and object weight.

**Figure 4:**
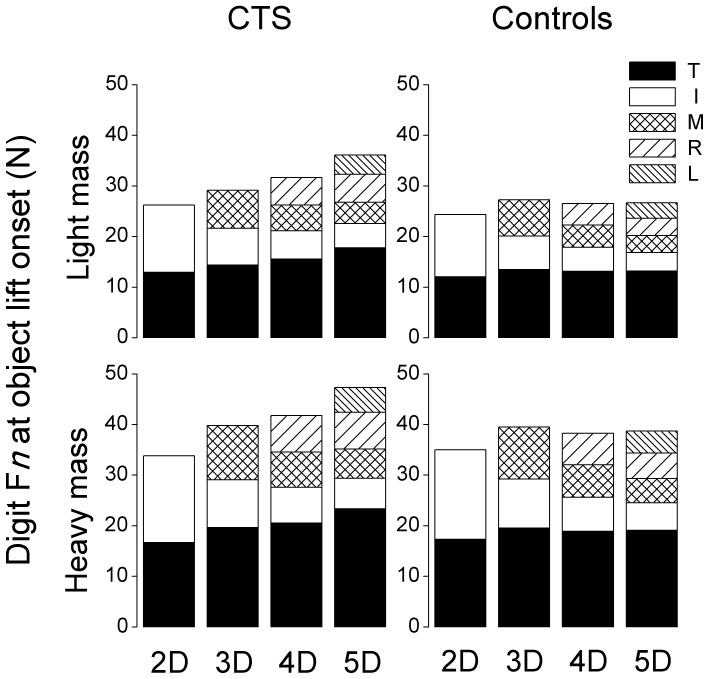
Individual digit normal forces at object lift onset. The normal force exerted by each digit at object lift onset is shown for each grip type, mass, and subject group (CTS and controls on the left and right column, respectively). Two-, three-, four- and five-digit grasps are denoted by 2D, 3D, 4D, and 5D, respectively. Data are mean values averaged across trials 3 through 7 for each subject group.

Figure 4 plots the individual F*n* averaged across subjects within each group and grip type (also see Table 3). As shown in the figure, the above interaction between grip type and group was caused by 1) above, i.e., the index and middle fingers performing similarly between the two groups across grip types and the ring and little fingers producing significantly larger F*n* in CTS patients in the 4D and 5D tasks regardless the object mass (main effect of *Group*: F_[1,30]_ = 6.34 and 4.16 for ring and little fingers, respectively; both *P*<0.05). For CTS-affected fingers (index and middle fingers), both groups reduced F*n* with each additional digit added to the grasp. Specifically, F*n* decreased from 2D to 3D, 3D to 4D, and 4D to 5D at the index finger (main effect of *Grip type*: F_[3,90]_ = 203.93, *P*<0.001), and decreased from 3D to 4D, and 4D to 5D at the middle finger (main effect of *Grip type*: F_[3,90]_ = 99.88, *P*<0.001). In contrast, the ring finger F*n* decreased when switching from 4D to 5D only in controls, whereas it remained constant in CTS patients (*Group × Grip type* interaction: F_[1,30]_ = 4.774, *P*<0.05). Both subject groups also increased F*n* at all digits when lifting the heavier object (main effect of *Mass*: F_[1,30]_ = 224.51, 113.36, 115.30, and 104.98 for thumb, index, middle, ring, and little fingers, respectively; all *P*<0.001).

**Table 3:** Individual Digits’ Normal Force (F*n*) and Tangential Force (F*tan*) at Object Lift Onset Averaged across Subjects within Each Group and Grip Type.

		Light mass				Heavy mass			
		2D	3D	4D	5D	2D	3D	4D	5D
**CTS F** ***n*** ** (N)**	T	12.9±1	14.4±1	15.6±1.7	17.8±1.8	16.7±1.2	19.6±1.3	20.5±2.1	23.3±2.4
	I	13.3±1	7.3±0.7	5.6±0.6	4.8±0.5	17.1±1.2	9.5±0.8	7.1±0.8	6.1±0.7
	M		7.5±0.5	5.1±0.7	4.2±0.5		10.7±0.8	6.9±0.9	5.8±0.7
	R			5.4±0.5	5.5±0.8			7.2±0.6	7.3±0.8
	L				3.8±0.4				4.9±0.5
**Controls F** ***n*** ** (N)**	T	12±0.8	13.5±0.6	13.1±1	13.2±0.8	17.3±1	19.5±0.9	18.9±1.1	19.1±0.8
	I	12.3±0.8	6.6±0.4	4.7±0.3	3.6±0.3	17.7±1.1	9.7±0.5	6.7±0.5	5.5±0.4
	M		7.1±0.4	4.4±0.5	3.4±0.3		10.3±0.6	6.4±0.6	4.7±0.4
	R			4.3±0.3	3.4±0.2			6.2±0.4	5.1±0.3
	L				3.1±0.3				4.3±0.4
**CTS F** ***tan*** ** (N)**	T	1.6±0.1	1.5±0.1	1.4±0.2	1.6±0.2	2.6±0.2	2.6±0.2	2.5±0.2	2.7±0.3
	I	1.7±0.1	0.4±0.1	0.1±0.2	−0.4±0.2	2.5±0.2	0.7±0.1	0.3±0.2	−0.2±0.2
	M		1.6±0.2	0.6±0.1	0.4±0.1		2.5±0.3	0.9±0.1	0.7±0.2
	R			1.3±0.2	1±0.2			2.1±0.2	1.4±0.2
	L				0.8±0.1				1.3±0.1
**Controls F** ***tan*** ** (N)**	T	1.8±0.1	1.8±0.1	2±0.1	1.9±0.2	3±0.2	3±0.1	3±0.2	3.3±0.2
	I	1.9±0.1	0.6±0.1	0.4±0.1	0.05±0.1	3±0.2	1.1±0.2	0.7±0.2	0.3±0.2
	M		1.5±0.1	0.6±0.1	0.4±0.1		2.2±0.1	1±0.1	0.7±0.1
	R			1.1±0.1	0.7±0.1			1.7±0.2	1±0.1
	L				0.8±0.2				1.3±0.2

Group differences were also found in across-trial variability of individual finger F*n* (CV_F*n*), with CTS patients exhibiting larger CV_F*n* at the middle and little fingers than controls (main effect of *Group*; middle finger: F_[1,30]_ = 6.55, *P*<0.05; little finger: F_[1,30]_ = 13.41, *P*<0.01). No significant interactions were found in CV_F*n*.

### Finger Tangential Force

In the present task, the resultant of all digits’ tangential forces (F*_T_*) at object lift onset has to be larger than, and during hold equal to, the object weight (Fig. 1B). Table 3 shows the average values of individual digits F*tan* at object lift onset across subjects within each group per grip type. All fingers contributed to generating larger F*tan* for the heavier object mass in both groups (main effect of *Mass*: F_[1,30]_ = 72.07, 115.10, 108.21, and 51.51 for index, middle, ring, and little fingers, respectively; all *P*<0.001). Furthermore, all subjects reduced individual finger F*tan* when more fingers were engaged in the grip (main effect of *Grip type*: F_[3,90]_ = 191.75, F_[2,60]_ = 96.21, F_[1,30]_ = 38.81 for index, middle, and ring fingers, respectively; all *P*<0.001). An important group difference was that CTS patients exerted significantly lower F*tan* at the index finger, but higher F*tan* at the ring finger compared with controls (main effect of *Group*: F_[1,30]_ = 5.69 and 5.85 for index and ring fingers, respectively; both *P*<0.05).

### Components of Compensatory Moment

**Figure 5:**
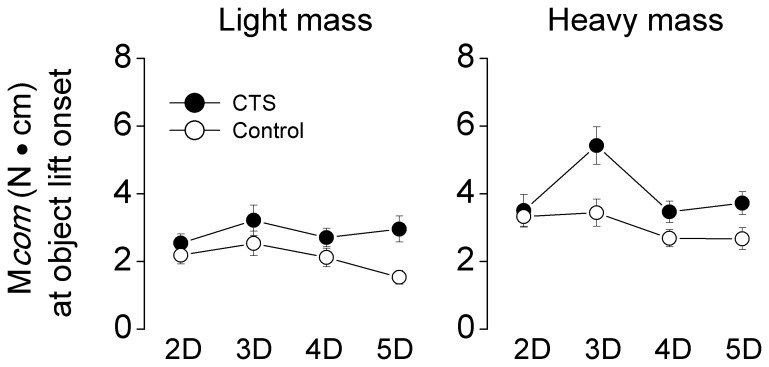
Compensatory moment at object lift onset and its components. The absolute value of the moment exerted on the object (M*com*) at object lift onset is shown for the light and heavy mass (left and right column, respectively) for each grip type and subject group. Data are mean values averaged across trials 3 through 7 for each subject group. Vertical bars denote standard errors.

The task requirement of ‘lift the object vertically’ required zero-moment production due to the object's symmetrical mass distribution. To evaluate subjects’ ability for anticipatory control of the net moment produced on the object at lift onset [9]–[10], the M*com* (Fig. 5) and its components (M*n* and M*tan*; see Methods) were analyzed for each grip type and mass condition. Regardless of object weight, both groups exhibited a small but non-zero M*com* at object lift onset which was larger for the heavier object and for the 3D grip type (main effect of *Mass*: F_[1,30]_ = 87.18, *P*<0.001; main effect of *Grip type*: F_[3,90]_ = 5.71, *P*<0.01). Note that, with the exception of the larger M*com* produced by the patients (5.4±0.55 N⋅cm) for the 3D grip in the heavy weight condition, both groups produced fairly consistent M*com* across grip types. Furthermore, the tendency for larger M*com* for the 3D grip in CTS than controls was not significant (no significant interaction *Group × Grip type*; *P* = 0.146). Importantly, CTS patients were less skilled than controls in minimizing the net moment (3.4±0.2 N⋅cm and 2.6±0.2 N⋅cm for CTS patients and controls, respectively) on the object at lift onset (Fig. 5; main effect of *Group*: F_[1,30]_ = 9.30, *P*<0.01), suggesting a diminished ability to coordinate M*n* and M*tan*. Specifically, this group difference was due to larger M*n* in CTS than controls (main effect of *Group*: F_[1,30]_ = 7.44, *P*<0.05), even though M*n* was partially cancelled by M*tan* that was produced in an opposite direction.

## Discussion

In the present study, we examined the effects of CTS on grasp control as a function of grip configuration when manipulating objects with different weights. The main findings are: CTS patients *(a)* exerted significantly larger grip force than controls but only for grips involving four and five digits; *(b)* were able to modulate multi-digit forces to object weight regardless of the number of digits involved in the grasp; and, consistent with previous findings, *(c)* were less able to coordinate multi-digit forces to minimize compensatory moment than controls for all grip types. These findings are discussed in the context of deficits in sensorimotor integration that might be specific to tasks involving the coordination of CTS-affected and non-affected digits.

### Multi-digit Force Modulation to Object Weight

Both CTS patients and controls increased grip force for lifting heavier objects in an anticipatory fashion, i.e., before object lift, as indicated by a modulation of peak grip force rate to object weight. Force modulation to object weight also persisted during object hold. Both findings are consistent with a recent study on five-digit grasping in CTS patients [9]. However, the current study extends this previous work by showing that CTS patients are able to modulate forces to object weight regardless of the number of digits involved in the grasp. Note that CTS patients’ ability for anticipatory control of multi-digit forces is found for grip types that involve only digits with impaired sensation (two- and three-digit grips) as well as these digits interacting with fully sensate digits (five-digit grip). Therefore, it is unlikely that grip force modulation to object weight reported by our previous work on five-digit grasping [9] is due only to intact cutaneous sensation from CTS non-affected digits. While our previous interpretation suggested that feedback from muscle, joint, and tendon mechanoreceptors in the forearm and upper arm - whose function is spared by median nerve compression – is integrated with residual somatosensory feedback from fingertips to infer object weight after the first object lift, the current results from grips performed with CTS-affected digits only suggest that the former source of feedback may play an even larger role.

### Grip-type Specific Effects of CTS on Grip Force

For a given object weight and frictional properties, the minimum grip force required to prevent object slip is invariant with respect to the number of digits involved in the grasp. However, the ability to exert the same total grip force across grip-types requires re-distributing normal force exerted by the digits. For example, the grip force exerted by adding a digit would have to be counterbalanced by a decrease in grip force in at least one digit. The fact that healthy controls used the same grip force across all grip types is evidence of efficient force coordination and control, and suggests that the intact CNS is not challenged by varying the number of digits in the grasp. In contrast, CTS patients produced grip-type specific differences in grip force with respect to controls. Specifically, CTS patients exerted similar grip force as controls when using only the CTS-affected digits (2D and 3D grips) to grasp and manipulate our grip device. This finding appears to contradict the work by Cole et al. [3] and Dun [4] based on acute compression and analgesia of the median nerve, respectively. However, both of these studies found excessive grip forces only under the condition of almost complete numbness of thumb and index finger, a condition not observed in the current CTS patients. In addition, the behavioral manifestations of acute changes may differ from changes in nerve function due to *chronic* compression as the latter may also lead to a reorganization of the somatosensory hand area in the cortex [17], [18] as well as a change in the excitability of the spinal cord [19].

Interestingly, patients exerted significantly larger normal forces when the more sensate digits, i.e., the ring finger, or the ring and little fingers, were added to the grip (Fig. 3). This was due to the CTS patients’ producing significantly larger normal forces in the ring and little fingers than the controls, which was not sufficiently compensated for by the reduction of normal forces at the index and middle fingers. The larger grip force exhibited by patients suggest that the effects of CTS on manipulation control are dependent on the number of digits engaged in the task, and more specifically that the combination of CTS-affected and non-affected digits leads to greater digit force coordination deficits than grips involving CTS-affected digits only. Note that patients had no difficulty in modulating digit forces when adding the middle finger to the grip, i.e., when changing grip type from 2D to 3D. Therefore, the challenge to multi-digit coordination does not appear to be related to just having more digits to coordinate. Furthermore, as multi-digit force coordination did not appear to be affected when only sensory impaired digits were used in the grasp (i.e., 3D grip), our results do not appear to be due to the CTS induced tactile deficits per se. In fact, we speculate that the grip-type dependent differences in multi-digit force coordination between CTS patients and controls are related to the *heterogeneity of tactile deficits* among the digits in CTS patients. Specifically, for grip types involving fewer digits (thumb and index, or thumb, index and middle fingers), sensation at all of these digits is reduced by median nerve compression. Conversely, four- and five-digit grips require coordination of digits that *are affected* with those *not affected* by CTS. It is therefore conceivable that the process of *integrating* intact and reduced sensory feedback from the fingertips might be more challenging than integrating feedback from CTS-affected digits only. This speculation leads to the prediction of elevated grip force for grip types involving CTS-affected and non-affected digits other than the two common grip types we studied, i.e., thumb and little finger, or thumb, ring and little fingers. While our interpretation requires further studies, it is supported by the finding that the larger grip forces produced by the CTS patients in the four- and five-digit grips were caused by adding the ring and little finger to a ‘default’ grip force sharing among the CTS-affected digits - thumb, index, and middle fingers (Fig. 4). The establishment of a ‘default’ force sharing pattern among these digits may further suggest that the congruence of impaired sensation from CTS-affected digits might have elicited a greater degree of long-term adaptation to reduced sensation and effectiveness in digit force coordination. Furthermore, evidence suggests that chronic CTS results in changes within the somatosensory system that has the potential to affect the activity of the ulnar nerve-innervated ring and little fingers. Specifically, Tinazzi et al. [20] observed increased sensory evoked potentials in the hand somatosensory cortex of CTS patients after stimulation of the *ulnar* nerve. How such changes in the sensory system with respect to ulnar nerve may affect the motor output of the ulnar nerve (i.e., ring and little finger) remains unknown.

### Biomechanical and Functional Considerations

The greater grip force associated with four- and five-digit grips in CTS patients can be viewed as an inefficient force control strategy as the ‘extra’ grip force does not add grasp stability, i.e., patients, like controls, could have used the same total grip force across all grip types to prevent object slip. Besides larger grip forces than controls for specific grip types, we also found that CTS were less able than controls in minimizing the net moment on the object, further suggesting a deficit in coordinating multi-digit forces. Interestingly, however, CTS patients’ decreased ability to minimize the net moment on the object with respect to controls did not vary as a function of grip type. This is a surprising result, given the grip-type specific force coordination deficits induced by CTS. Most importantly, this finding indicates that the larger grip force associated with four- and five-digit grips was effectively compensated for to maintain the non-zero moment relatively constant across grip types (Fig. 5). In turn, this compensation implies a residual, but incomplete, ability in CTS patients to shift net center of pressure by re-distributing normal forces, and further suggests that such ability might be independent of CTS’ inability to finely modulate force magnitude. This interpretation is consistent with evidence showing an intact ability in CTS patients to shift finger center of pressure by altering the normal force distribution as a function of object mass distribution [10], a phenomenon that is likely implemented through CTS-spared extrinsic finger muscles.

### Conclusions

The present findings confirm the previous observation of CTS patients’ residual ability to modulate multi-digit forces to object weight in whole-hand grasping and extend it to all other grip types. We also confirmed the finding of CTS patients’ reduced ability to minimize the net moment on the object. However, the most important finding was that CTS patients exhibited grip type-specific force coordination deficits as they exerted larger grip force when CTS non-affected digits had to be coordinated with CTS-affected digits. This novel finding suggests that the integration of intact and reduced sensory feedback from the fingertips might challenge the central nervous system to a greater degree than integrating feedback from CTS-affected digits only. More work is needed to address the extent to which sensorimotor coordination deficits described by the present study might be exacerbated in patients affected by a greater CTS severity.
